# P-1302. Alterations in the population structure and antimicrobial resistance of *Salmonella enterica* before and during the COVID-19 pandemic (2018 and 2020) in New York State

**DOI:** 10.1093/ofid/ofae631.1483

**Published:** 2025-01-29

**Authors:** Jose A Rodrigues, Odion O Ikhimiukor, Stephanie S R Souza, Matthew Marmion, Lisa Mingle, Danielle Wroblewski, Samantha E Wirth, Kimberlee A Musser, William J Wolfgang, Cheryl P Andam

**Affiliations:** State University of New York, Columbia University Irving Medical Center, New York, New York; University at Albany, State University of New York, Albany, New York; University at Albany, State University of New York, Albany, New York; University at Albany, State University of New York, Albany, New York; Wadsworth Center, New York State Department of Health, Albany, New York; Wadsworth Center, New York State Department of Health, Albany, New York; Wadsworth Center, New York State Department of Health, Albany, New York; Wadsworth Center, New York State Department of Health, Albany, New York; Wadsworth Center, New York State Department of Health, Albany, New York; University at Albany, State University of New York, Albany, New York

## Abstract

**Background:**

The COVID-19 pandemic impacted many core activities to combat antimicrobial resistance (AMR). Concurrently, there has been increased AMR, particularly in hospital-acquired infections. However, there is little data on AMR surveillance of community-acquired infections, such as foodborne infections, during this period. An estimated 212,500 resistant *Salmonella enterica* infections occur yearly. Here, we aim to elucidate the impact of the COVID-19 pandemic on the population dynamics of *S. enterica* lineages and the genetic determinants of AMR.

**Figure 1:**
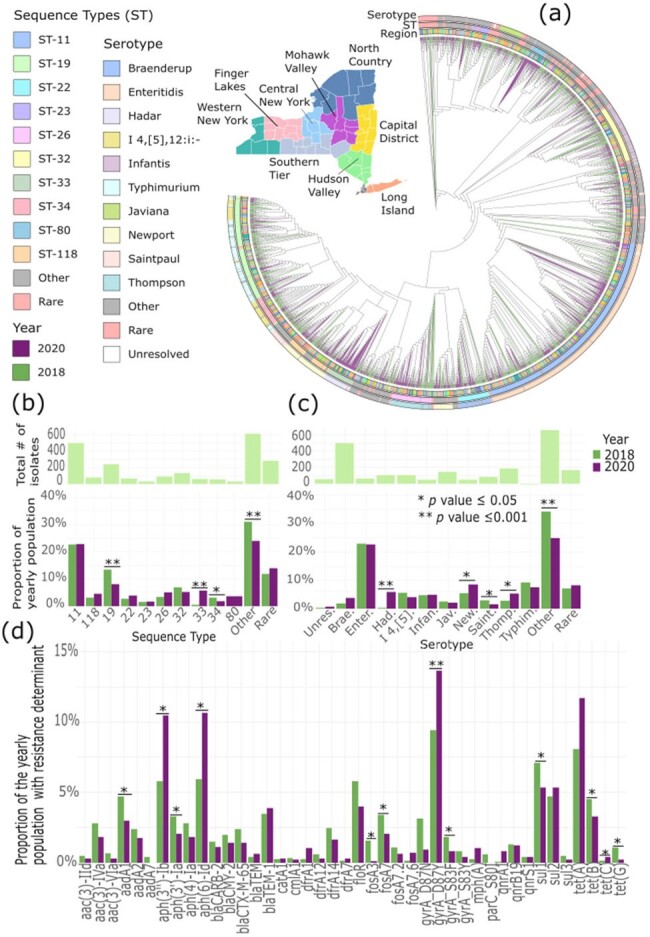
Phylogenetic relationships, pan-genomic, lineage and antimicrobial resistance characteristics of [i]S. enterica[i] genomes before (2018, n=1,212) and during the COVID-19 pandemic (2020, n=975) A. A midpoint-rooted maximum likelihood phylogenetic tree based on 608,598 core-gene single nucleotide polymorphisms. Branch lengths are not displayed. Outer rings show Serotype, Sequence Type, and Region. Branches are colored by sampling year. A map displays the region associated with the source county of the isolate within New York State. B. Displays the Sequence Types as the proportion of the total population. Chi-squared p-values are displayed. C. Serotype as proportions of the yearly population. D. Resistance mechanism as a proportion of the yearly population Benjamini-Hochberg corrected p-values are displayed.

**Methods:**

We leveraged 2,187 publicly available sequences derived from *S. enterica* isolates from human clinical samples across 56 counties in New York, USA, before the pandemic (2018, n=1,212 genomes) and during the pandemic (2020, n=975 genomes).

**Results:**

A midpoint-rooted maximum likelihood phylogeny generated from 608,598 single-nucleotide polymorphisms from 3,482 core genes revealed the intermingling of isolates regardless of year and county of isolation. We did not find significant differences in the diversity (measured using the Simpson diversity index) of sequence types (ST) and serotypes between the two time periods. However, some STs and serotypes exhibited significant changes in the proportion of the annual population. We observed an increase in ST33 and a reduction in ST19, ST34, and low-frequency STs. There was a marked increase in serotypes Hadar, Newport, and Thompson and a decrease in serotypes Saintpaul and the low-frequency serotypes. We also measured the number of genomes carrying specific AMR genes as a proportion of the annual population per year. We found a significant change in the number of genomes carrying in aph(3”)-Ib and aph(6”)-Id (aminoglycoside resistance), tetC (tetracycline), and the fluoroquinolone resistance point mutation (gyrA_D87Y).

**Conclusion:**

Our findings revealed the bacterial features, such as locally circulating lineages, mutations, and mobile antimicrobial resistance genes, that were altered before and during the COVID-19 pandemic. Together, our data show that major public health emergencies such as the COVID-19 pandemic can inadvertently alter the dynamics of other pathogen populations, including foodborne bacteria, which may have consequences for public health and treatment efforts.

**Disclosures:**

**All Authors**: No reported disclosures

